# Synthesis of 2,3,5,6-tetrafluoro-pyridine derivatives from reaction of pentafluoropyridine with malononitrile, piperazine and tetrazole-5-thiol

**DOI:** 10.1186/s40064-015-1495-4

**Published:** 2015-12-04

**Authors:** Khalil Beyki, Reza Haydari, Malek Taher Maghsoodlou

**Affiliations:** Department of Chemistry, Faculty of Science, University of Sistan and Baluchestan, P. O. Box 98135-674, Zahedan, Iran

**Keywords:** Pentafluoropyridine, Heterocycle, Nucleophilic Substitution, Synthesis, ^19^F-NMR

## Abstract

Some pentafluoropyridine derivatives have been synthesized by the reaction of pentafluoropyridine with appropriate C, S and N-nucleophile such as malononitrile, 1-methyl-tetrazole-5-thiol and piperazine. These reactions provided 4-substituted 2,3,5,6-tetrafluoropyridine derivatives in good yields. All the compounds were characterized using ^1^H, ^13^C and ^19^F-NMR spectroscopy and X-ray crystallography.

## Background

Pentafluoropyridine and related compounds in which all the hydrogen atom in heterocyclic ring have been replaced by fluorine atoms were synthesized by reaction of potassium fluoride with perchloro heteroaromatic (Ojima 2009). In pharmacology, it is common to substitute hydrogen with fluorine atoms for increases the lipophilicity and biological activity of the compounds (Chambers et al. 2008a, b). Pentafluoropyridine one of the most important perfluoroheteroaromatic compounds have been used for the synthesis of various drug-like systems (Gutov et al. 2010). These systems are highly active towards nucleophilic additions owing to the presence of electronegative fluorine atoms and the presence of the nitrogen heteroatom so all five fluorine atoms in pentafluoropyridine may be substituted by an appropriate nucleophile (Cartwright et al. 2010; Chambers et al. 2005). A nucleophilic substitution reaction of pentafluoropyridine occurs in two-step addition–elimination mechanism, so install nucleophile addition and in the end elimination flour ring nitrogen (Colgin et al. 2012). The site reactivity order of pentafluoropyridine is well known that, the order of activation toward nucleophilic attack follows these quence 4 (*Para*)-fluorine > 2 (*Ortho*)-fluorine > 3 (*Meta*)-fluorine so the reactions of pentafluoropyridine with some nucleophilic occur selectively at the *Para* position as this site is most activated towards nucleophilic additions to afforded of 4-substited tetrafluoropyridine (Chambers et al. 2008a, b).

## Results and discussion

**Fig. 1:**
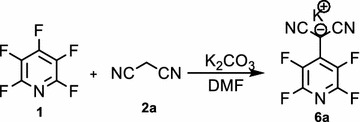
Reaction of pentafluoropyridine with malononitrile

In this research, we describe nucleophilic substitution of pentafluoropyridine with a wide range of nucleophiles and highlight how the resulting products 4-substited-2,3,5,6-tetrafluoro-pyridine derivatives. Reaction of pentafluoropyridine **1** with malononitrile **2a** under basic conditions (K_2_CO_3_) in DMF at reflux gave a 4-(malononitrile)-2,3,5,6-tetrafluoropyridine **6a** (Fig. 1).

**Fig. 2:**
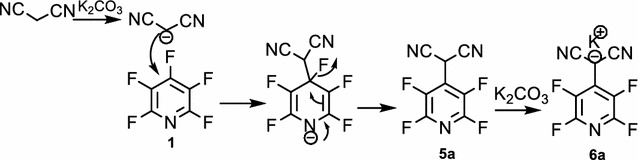
The suggested mechanism nucleophilic substitution of pentafluoropyridine with malononitrile

**Fig. 3:**
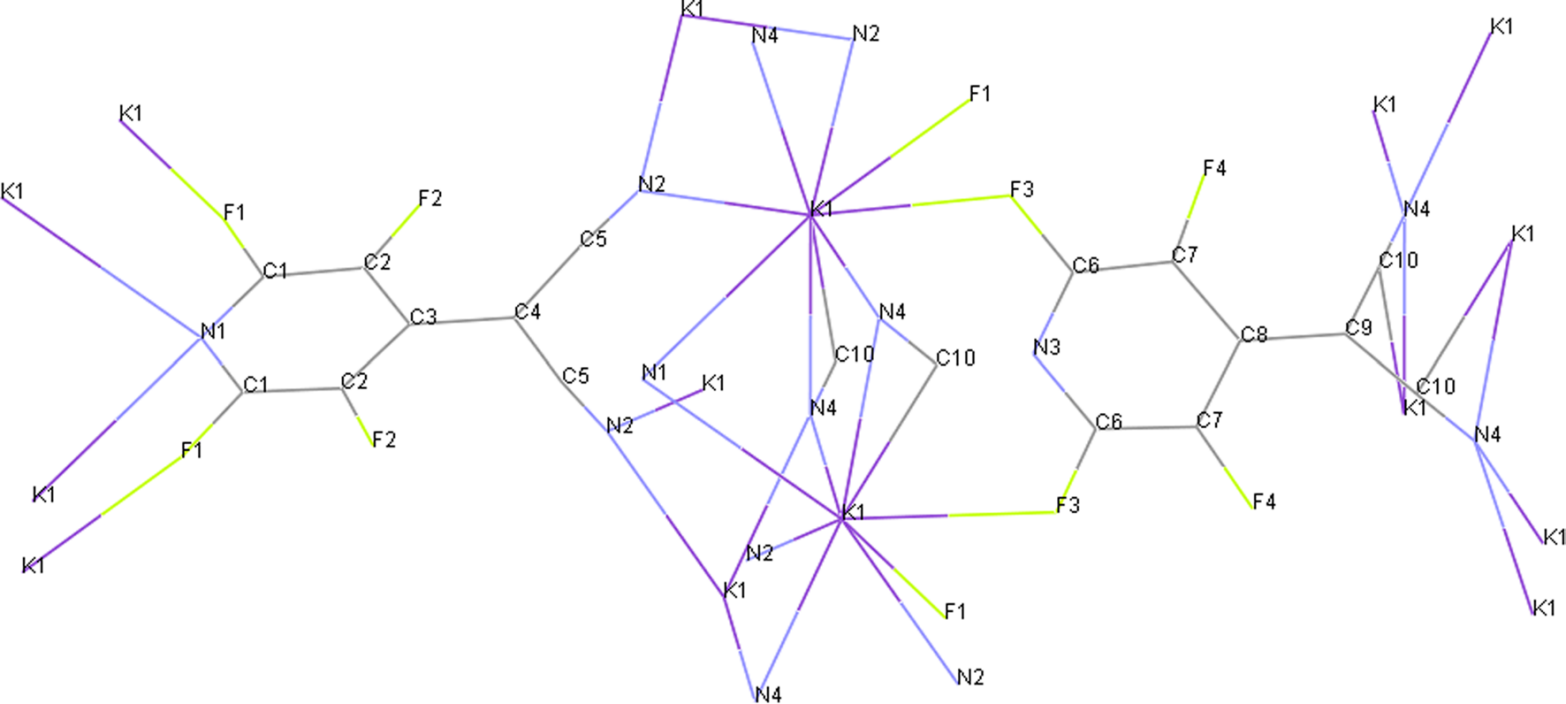
X-ray structure of **6a**

**Fig. 4:**
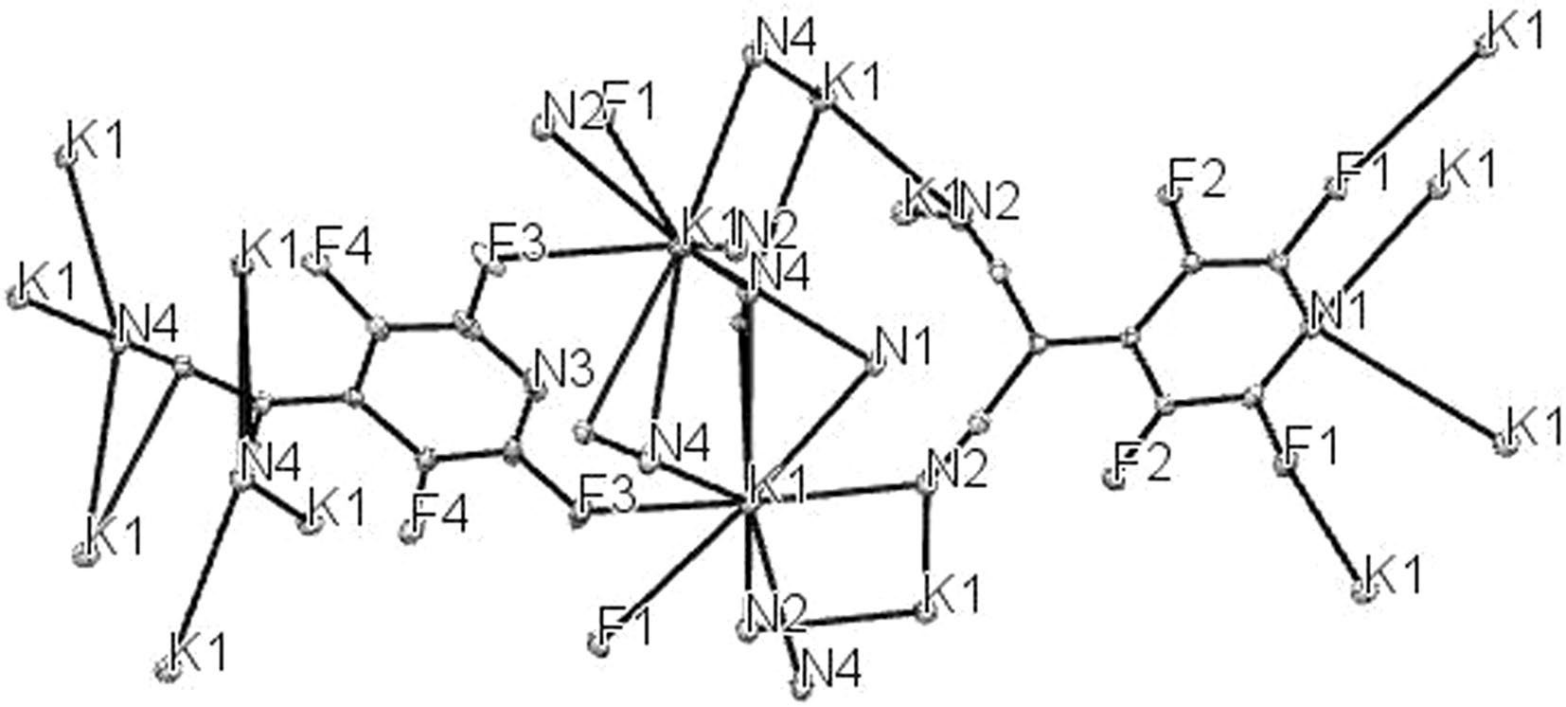
ORTEP diagram of **6a**

In basic condition, malononitrile **2a** deprotonate and carbon nucleophile of malononitrile attack to *Para* position of pentafluoropyridine **1** and elimination of 4-fluor ring pyridine to give **5a**. In **5a**, hydrogen malononitrile very acidy so essay deprotonate in base solution to give potassium dicyano (perfluoropyridin-4-yl)methanide **6a** (Fig. 2). Purification of **6a** was achieved by recrystallization in ethanol/acetonitrile. In crystal **6a**, two molecule chelate by potassium ion between flour and nitrogen. Identification of chelate **6a** was done by ^19^F-NMR analysis, in which the resonance attributed to fluorines located *Ortho* to ring nitrogen has a chemical shift of -83.5 ppm and -84.4 ppm. The corresponding resonance for fluorines located *Meta* to ring nitrogen in chelate **6a** occurred at −135.4 and −139.4 ppm. Four resonances by ^19^F-NMR indicate displacement of fluorine atoms attached to the *Para* position of two pyridine ring. The ^1^H-NMR spectra of compound **6a** consisted of a H broad signal at δ = 7.29 ppm for CH malononitrile. X-ray crystallography confirmed the structure of chelate **6a** (Figs. 3, 4). A summary of the crystal data, experimental details and refinement results for **6a** is given in Table 1.

**Table 1:** Crystal data for **6a**, **5b** and **3c**

Compound	**6a**	**5b**	**3c**
Formula	C_8_ F_4_ K N_3_	C_9_ H_8_ F_3_ N_5_ O S	C_14_ H_8_ F_8_ N_4_
Formula weight	253.21	291.26	384.24
Wavelength	0.71073	0.71073	0.71073
Crystal system	Monoclinic	Monoclinic	Orthorhombic
Space group	C2/c	P 21/n	P b c a
Unit cell dimensions (Å)	*a* = 11.882 (2) *b* = 18.857 (4) *c* = 7.7561 (15) *α* = 90 *β* = 108.369 (3) *γ* = 90	*a* = 9.0254 (9) b = 7.5269 (8) c = 17.9941 (19) *α* = 90 *β* = 99.1260 (10) *γ* = 90	*a* = 8.8425 (5) *b* = 11.0779 (4) *c* = 14.5459 (7) *α* = 90 *β* = 90 *γ* = 90
Volume Å^3^	1649.2 (6)	1206.9 (2)	1424.86 (12)
Z	8	4	4
Density (calculated) g cm^−1^	2.040	1.603	1.791
F(000)	992	592	768
Crystal size	0.469 × 0.196 × 0.165	0.309 × 0.240 × 0.151	–
*Θ* range for data	2.99°–32.57°	0.999°–1.000°	3.264°–28.311°
Index range	−17 < h < 17 −28 < k < 28 −11 < l < 11	−12 < h < 12 −10 < k < 10 −25 < l < 25	−11 < h < 11 −14 < k < 13 −19 < l < 18
Absorption coefficient mm^−1^	0.682	0.307	0.184
Parameters/restraints	148/0	0	0
Final *R*_*1*_^*a*^ *. wR*_*2*_^*b*^ (Obs. data)	0.0249, 0.0781	0.0503, 0.0411	0.0500, 0.1435
Final *R*_*1*_^*a*^, *wR*_*2*_^*b*^ (all data)	0.0277, 0.0781	0.1207, 0.1122	0.0690, 0.1681
Goodness of fit on *F* ^2^ (S)	1.468	1.035	1.295

**Fig. 5:**
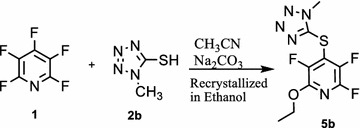
Reaction of pentafluoropyridine with 1-methyl-tetrazole-5-thiol

**Fig. 6:**
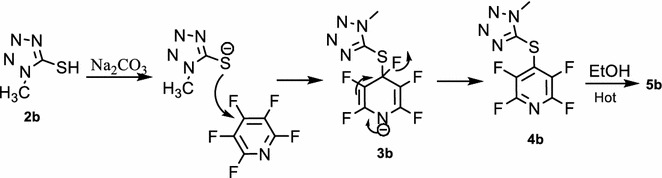
The suggested mechanism nucleophilic substitution of pentafluoropyridine with 1-methyl-1H-tetrazole-5-thiol

**Fig. 7:**
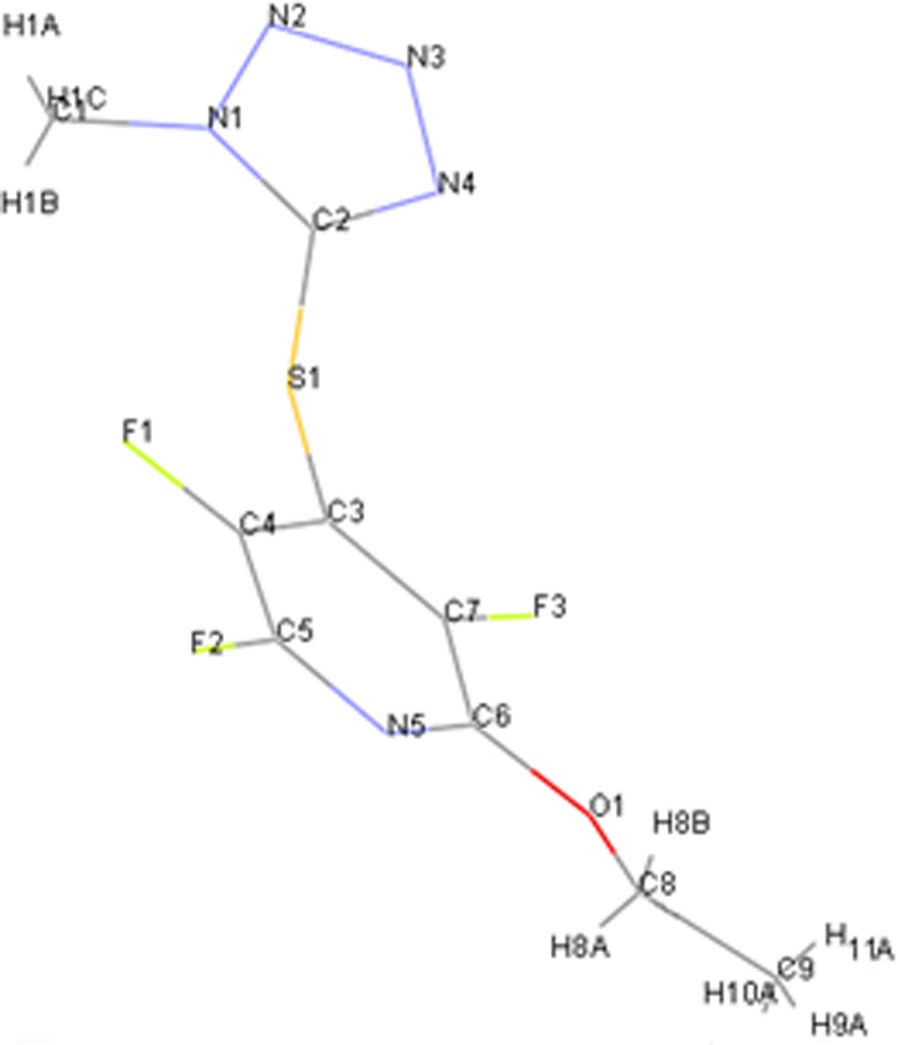
X-ray structure of **5b**

**Fig. 8:**
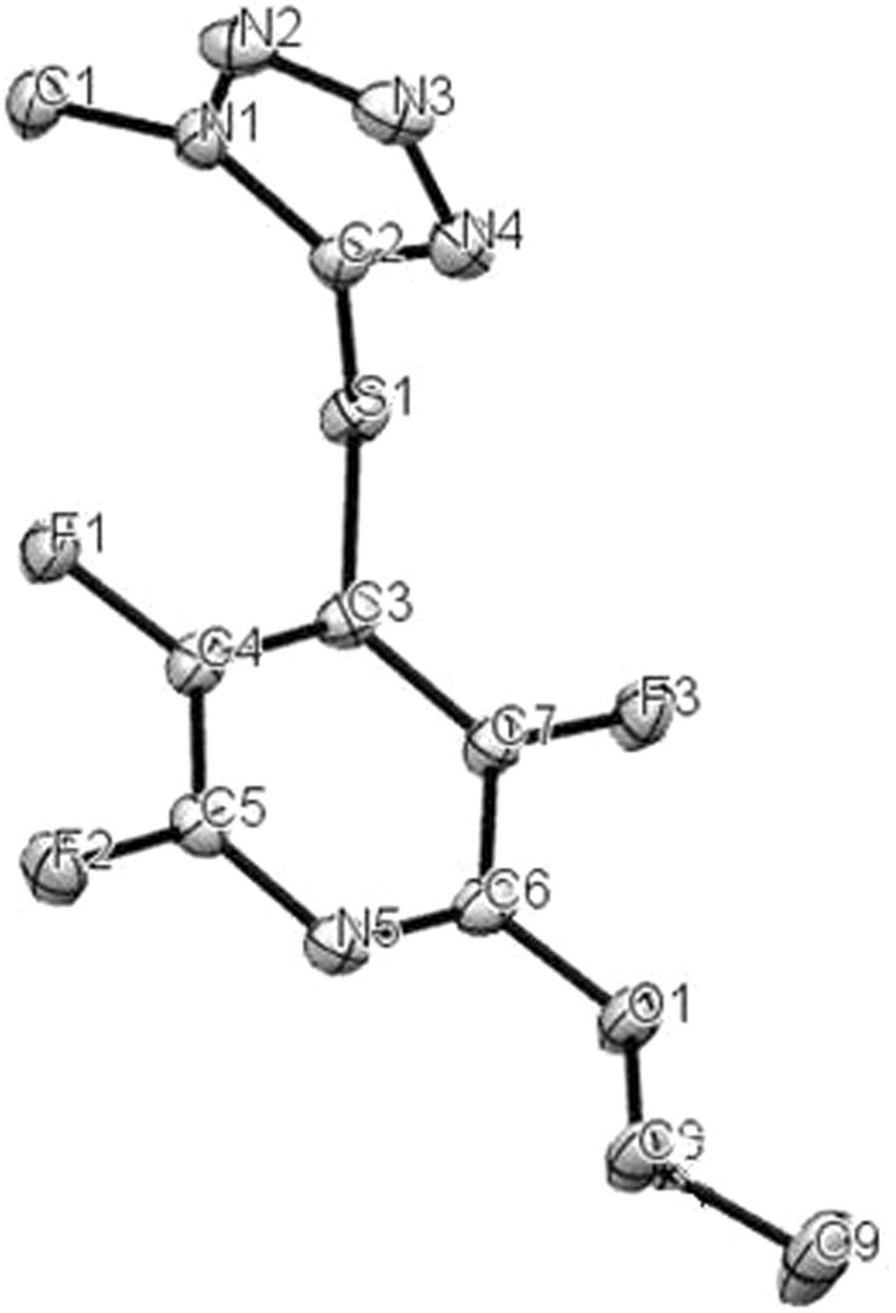
ORTEP diagram of **5b**

Reaction of 1-methyl-tetrazole-5-thiol **2b** with pentafluoropyridine **1** in acetonitrile at reflux temperature and recrystallisation in ethanol gave 2-ethoxy-3,5,6-trifluoro-4-((1-methyl-1H-tetrazol-5-yl)thio)pyridine **5b** (Fig. 5). In 1-methyl-1H-tetrazole-5-thiol, sulfur atom more nucleophilic than other atoms, so install attack at the *Para* position of the pyridine ring to give **4b**. Purification of **4b** was achieved by recrystallization in ethanol (accessible and non-toxic solvent). In hot EtOH, Ethoxy group attack at *ortho* position of 2,3,5,6-tetrafluoro-4-(1-methyl-1H-tetrazol-5-ylthio)pyridine **4b** to give **5b** (Fig. 6). Identification of **5b** was done from ^19^F-NMR analysis in which the resonance attributed to displacement of fluorine atoms attached only at the *Para* and *Ortho* position of the pyridine ring. The corresponding resonance for F-3,5 (*Meta*) in **5b** occurs at -131 and -154 ppm and F-6 (*ortho*) at −88 ppm. Other spectroscopic techniques were consistent with the structures proposed. The protons of the methyl group, were observed at δ = 4.13 ppm. The molecular structure of the 2-ethoxy-3,5,6-trifluoro-4-((1-methyl-1H-tetrazol-5-yl)thio) pyridine obtained has been determined by X-ray crystallography (Figs. 7, 8). A summary of the crystal data, experimental details and refinement results for **5b** is given in Table 1.

**Fig. 9:**
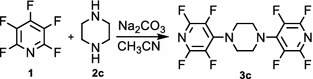
Reaction of pentafluoropyridine with piperazine

**Fig. 10:**
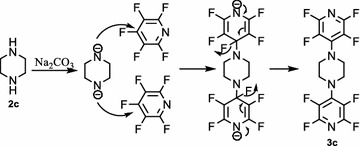
The suggested mechanism nucleophilic substitution of piperazine with pentafluoropyridine

**Fig. 11:**
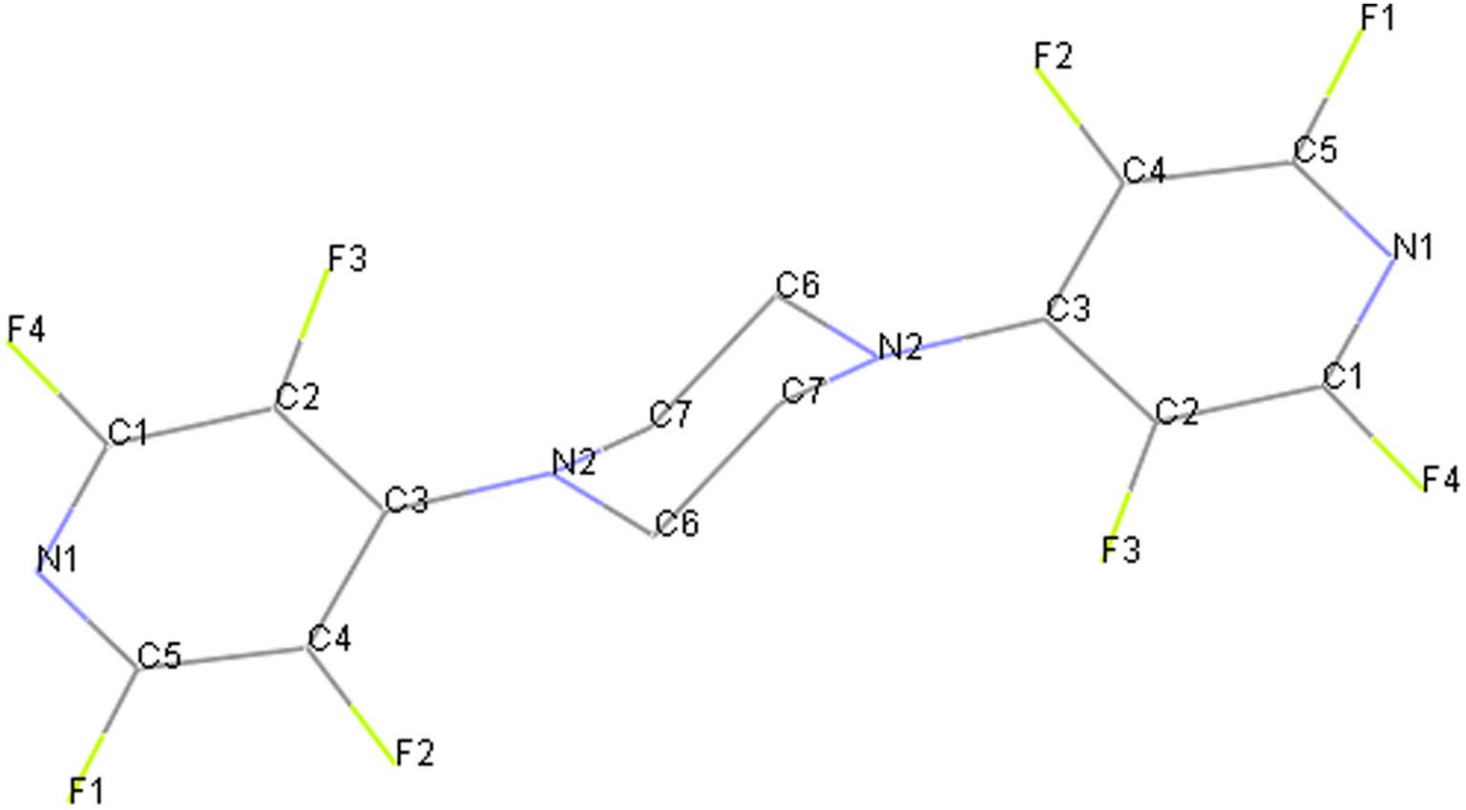
X-ray structure of **3c**

**Fig. 12:**
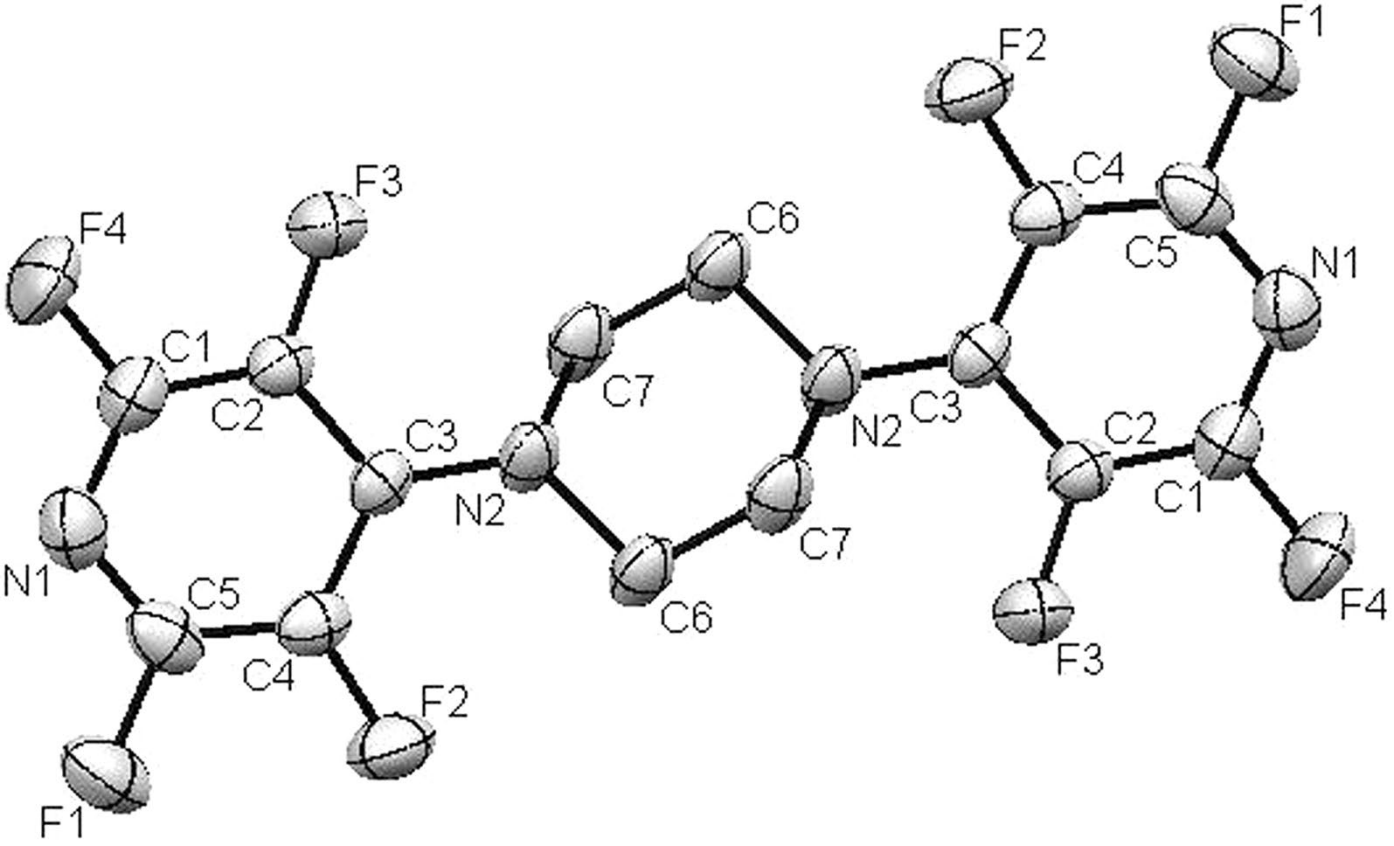
ORTEP diagram of **3c**

Also, we examined the reaction of pentafluoropyridine **1** with piperazine **2c** in the presence of sodium carbonate in CH_3_CN solvent gave 1,4-bis(perfluoropyridin-4-yl)piperazine **3c** (Fig. 9). In basic condition, two nitrogen of the piperazine deprotonation and attack to *Para* position of pentafluoropyridine and elimination of 4-fluor pyridine ring to give 3e (Fig. 10). Purification of **3c** was achieved by recrystallization in acetonitrile. The structure of compounds **3c** was confirmed by X-ray crystallography and by NMR spectroscopic data. In particular, ^19^F-NMR spectroscopy shows the chemical shift of fluorine atoms attached to the *Ortho* and *Meta* position are observed respectively at −97.3 and −160.5 ppm. In ^1^H-NMR, the protons of CH_2_ piperazine, was observed at δ = 4.3 ppm. The ^13^C-NMR spectrum of compound **3c** showed 4 distinct resonances in agreement with the proposed structure. The structure of **3c** was confirmed by X-ray crystallography (Figs. 11, 12).

## Conclusion

In conclusion, we showed that pentafluoropyridine can successfully react with a variety of nucleophiles to afford of 4-substited tetrafluoropyridine. The regioselectivity of nucleophilic substitution in this process may be explained by high nucleophilicity of sulfur, nitrogen or oxygen and activating influence of pyridine ring nitrogen that significantly activate the Para and Ortho sites to itself.

## Experimental

All materials and solvents were purchased from Merck and Aldrich and were used without any additional purification. The melting points of the products were determined in open capillary tubes using BAMSTEAB Electrothermal apparatus model 9002. The 1H NMR spectra were recorded at 300 MHz. The ^13^C-NMR spectra were recorded at 75 MHz. The ^19^F-NMR spectra were recorded at 282 MHz. In the ^19^F-NMR spectra, up field shifts were quoted as negative and referenced to CFCl_3_. Mass spectra were taken by a Micro mass Platform II: EI mode (70 eV). Silica plates (Merck) were used for TLC analysis.

### Preparation of 2-(perfluoropyridin-4-yl)malononitrile **6a**

Pentafluoropyridine **1** (0.1 g, 0.6 mmol), malononitrile **2a** (0.04 g, 0.6 mmol) and potassium carbonate (0.11 g, 1.0 mmol) were stirred together in DMF (5 mL) at reflux temperature for 3 h. The reaction mixture was evaporated to dryness than the solid product was recrystallisation from acetonitrile to give 2-(perfluoropyridin-4-yl)malononitrile (0.22 g, 86 %) as a red crystals; mp 260 °C dec, ^19^F NMR (acetone): ^1^H NMR (acetone): δ (ppm) 7.79 (s, 1H, CH); δ (ppm) −83.5 (m, 2F, F-2,6), −84.4 (m, 2F, F-2′,6′), −135.4 (m, 2F, F-3,5), −139.4 (m, 2F, F-3′,5′). MS (EI), m/z (%) = 508 (M^+^), 440, 364, 291, 180, 147, 121, 105, 91, 77, 57, 43.

### Preparation of 2-ethoxy-3,5,6-trifluoro-4-((1-methyl-1H-tetrazol-5-yl)thio)pyridine **5b**

Pentafluoropyridine **1** (0.1 g, 0.6 mmol), 1-methyl-1H-tetrazole-5-thiol **2b** (0.09 g, 0.6 mmol) and sodium hydrogencarbonate (0.11 g, 1.0 mmol) were stirred together in CH_3_CN (5 mL) at reflux temperature for 4 h (monitored by TLC). The solvent was evaporated; water (5 mL) was added and extracted with dichloromethane and ethyl acetate (3 × 5 mL). Solvent evaporation and recrystallisation from ethanol gave 2-ethoxy-3,5,6-trifluoro-4-((1-methyl-1H-tetrazol-5-yl)thio)pyridine **5b** (0.2 g, 75 %) as a white crystal; mp 130 °C dec. ^1^H NMR (acetone): δ (ppm) 1.37 (3H, m, CH_3_), 3.90 (3H, s, N-CH_3_), 4.3 (2H, m, CH_2_); ^19^F NMR (acetone): δ (ppm) −88.6 (1F, m, F-2), −131.4 (1F, m, F-3), −154.8 (1F, m, F-5); ^13^C NMR (acetone): δ (ppm) 14.6, 35.5, 63.2, 64.4, 139.2, 140.5, 142.6, 143.7, 145.9 ppm. MS (EI), m/z (%) = 292 (M^+^), 263, 235, 219, 180, 132, 100, 83, 43.

### Preparation of 1,4-bis(perfluoropyridin-4-yl)piperazine **3c**

Pentafluoropyridine **1** (0.1 g, 0.6 mmol), piperazine **2c** (0.03 g, 0.5 mmol) and sodium hydrogencarbonate (0.11 g, 1.0 mmol) were stirred together in CH_3_CN (5 mL) at reflux temperature for 5 h. After complicated reaction, the solvent was evaporated; water (5 mL) was added and extracted with dichloromethane and ethyl acetate (3 × 5 mL). Solvent evaporation and recrystallization from CH_3_CN gave 1,4-bis(perfluoropyridin-4-yl)piperazine **3c** (0.2 g, 52 %) as a white crystal; mp 288 °C dec. ^1^H NMR (acetone): δ (ppm) 4.30 (8H, s, CH_2_); ^19^F NMR (acetone): δ (ppm) −97.3 (4F, m, F-2,6), −160.5 (4F, m, F-3,5). ^13^C-NMR (acetone): δ (ppm) 60.3, 123.7, 127.1, 131.3 ppm. MS (EI), m/z (%) = 384 (M^+^), 317, 292, 263, 235, 219, 180, 152, 132, 116, 100, 83, 63, 43.
